# Experimental Investigation on the Application of Polymer Agents in Offshore Sandstone Reservoirs: Optimization Design for Enhanced Oil Recovery

**DOI:** 10.3390/polym17050673

**Published:** 2025-03-02

**Authors:** Yanyue Li, Changlong Liu, Yaqian Zhang, Baoqing Xue, Jinlong Lv, Chuanhui Miao, Yiqiang Li, Zheyu Liu

**Affiliations:** 1 College of Petroleum Engineering, China University of Petroleum (Beijing), Beijing 102249, China; 2 Tianjin Branch of CNOOC (China) Co., Ltd., Tianjin 300459, China; 3 State Key Laboratory of Offshore Oil and Gas Exploitation, Beijing 102209, China

**Keywords:** strong heterogeneity, polymer agent, deep profile control, polymer slug combination

## Abstract

The conventional polymer gel has high initial viscosity and short gelation time, making it difficult to meet the requirements of deep profile control in offshore reservoirs with large well spacing and strong heterogeneity. This paper evaluates the performance and core plugging capacity of novel functional polymer gels and microspheres to determine the applicability of core permeability ranges. On the heterogeneous core designed based on the reservoir characteristics of Block B oilfield, optimization was conducted separately for the formulation, dosage, and slug combinations of the polymer gel/microsphere. Finally, oil displacement experiments using polymer and microsphere combinations were conducted on vertically and planar heterogeneous cores to simulate reservoir development effects. The experimental results show the novel functional polymer gel exhibits slow gelation with high gel strength, with viscosity rapidly increasing four days after aging, ultimately reaching a gel strength of 74,500 mPa·s. The novel functional polymer gel and polymer microsphere can effectively plug cores with permeabilities below 6000 mD and 2000 mD, respectively. For heterogeneous cores with an average permeability of 1000 mD, the optimal polymer microsphere has a concentration of 4000 mg/L and a slug size of 0.3 PV; for heterogeneous cores with an average permeability of 4000 mD, the optimal functional polymer gel has a concentration of 7500 mg/L and a slug size of 0.1 PV. In simulations of vertically and planarly heterogeneous reservoirs, the application of polymer agent increases the oil recovery factor by 53% and 38.7% compared to water flooding. This realizes the gradual and full utilization of layers with high, medium, and low permeability.

## 1. Introduction

In 2022, China’s total offshore crude oil production surpassed 50 million tons [1,2], accounting for approximately 25% of domestic crude oil production [3]. This represents a significant guarantee for the stable and increased production of oil and gas in China. However, the complexity of reservoir types [4,5], large reservoir thickness [6], heterogeneity [7,8], injection rates [9], well trajectory [9], etc., has exacerbated contradictions within and among layers during long-term waterflooding development. The development of preferential flow paths and severe water channeling has severely constrained the effectiveness of oilfield development [10,11,12,13,14]. Practice has proven that chemical profile-control technology has become an important technical means to improve the effectiveness of waterflooding development in offshore oilfields, laying a solid foundation for stable and increased production in waterflooded offshore oilfields [15,16,17,18].

Currently, the most common polymer gel is the polyacrylamide (HPAM)/chromium crosslinked gel agent [19,20,21,22]. Owing to its inherent properties and environmental factors, this gel agent exhibits high initial viscosity (>80 mPa·s) and short gelation time, leading to a rapid increase in injection pressure during injection. This can easily contaminate or block low- and medium-permeability layers, making it difficult to migrate to the deep oil layer and only plug high-permeability layers [23,24,25,26,27]. Core simulation experiments by Sorbie [28,29] indicate that when the initial viscosity of polymer gel agents exceeds 20 mPa·s, the amount of polymer entering medium- and low-permeability layers is approximately 84% of that entering high permeability layers, failing to achieve deep profile control in the oil layer. Therefore, new chemical agents suitable for offshore reservoirs are needed that can migrate to the deep formations and effectively plug high-permeability layers.

To enable the gel to migrate to the deep reservoir and produce effective plugging, Wang [30], Huang [31], and Song [32] proposed a controllable gel with a long gelation time, high gel strength, and low initial viscosity. Cheng Jiecheng [33] and Wang Fei [34] used functional polymer gel to plug high-permeability layers with water channeling, clarifying their deep migration capabilities in heterogeneous cores and achieving better water reduction and oil recovery increase effects in field implementations. Xia Junyong [35] and Zhang Weisen [36] determined the concentration range of the gel system components to meet the profile control strength requirements of onshore oilfields and designed an optimal slug size of 0.1 PV using three-tube parallel experiments. Man Bo optimized the parameters of the subsequent polymer solution for functional polymer gel [37]. Although the functional polymer gel has been applied in field practices for deep profile control onshore, and scholars have studied the profile control mechanism, migration depth, and slug size of the low initial viscosity system, no scholars have yet provided a scheme design and development effect for this slowly gelled, deep profile control, and high-strength agent in complex reservoirs.

In response to this, this paper designs cores based on the characteristics of offshore reservoirs, conducts comparative experiments on the gelation performance of a new functional polymer gel versus conventional gels, and optimizes the gelation time, gel strength of agents at different concentrations. Tests on the injectability and plugging capacity of the functional polymer gel and polymer microsphere were conducted, along with experimental studies on the effects of agent concentration, agent size, and reservoir heterogeneity on the effectiveness of polymer flooding. These studies aim to determine the applicable range of functional polymer gel and polymer microsphere in sandstone reservoirs and the oil displacement effect under different factors. The optimal combination scheme for functional polymer gel and polymer microsphere in heterogeneous reservoirs is proposed to provide theoretical guidance and technical support for field implementation.

## 2. Experimental Section

### 2.1. Materials and Instruments

#### 2.1.1. Preparation of Chemical Agents

Functional polymer gel and polymer microsphere were used in the experiment. The effective concentration of the agents was 35% (all provided by COSL).

**Figure 1:**
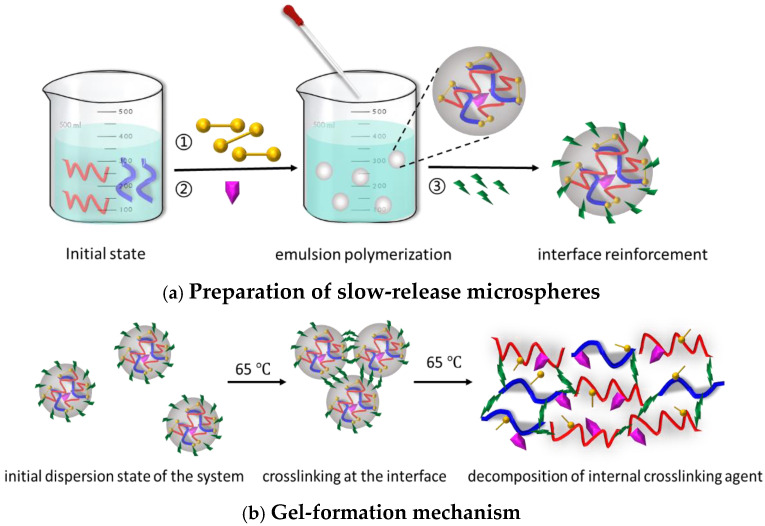
Preparation and Gelation Mechanism of Functional polymer gel.

The functional polymer gel was prepared using a low-temperature inverse emulsion polymerization method, as shown in Figure 1a. The decomposable crosslinking agent was first coated in the core layer of polymer microspheres, and then the organic crosslinking agent (MBA) was polymerized in the polymer shell layer to form highly dispersed slow-release profile-control microspheres. Due to their high dispersion, the system exhibited low initial viscosity. Under 65 °C, the organic crosslinking agent at the interface of the slow-release profile control microspheres crosslinked, forming aggregates between the microspheres, with a slight increase in viscosity. As time increased, the decomposable crosslinking agent inside the aggregates decomposed, forming a strong spatial network structure, and the system viscosity rapidly increased, as shown in Figure 1b.

#### 2.1.2. Core Model Design

**Figure 2:**
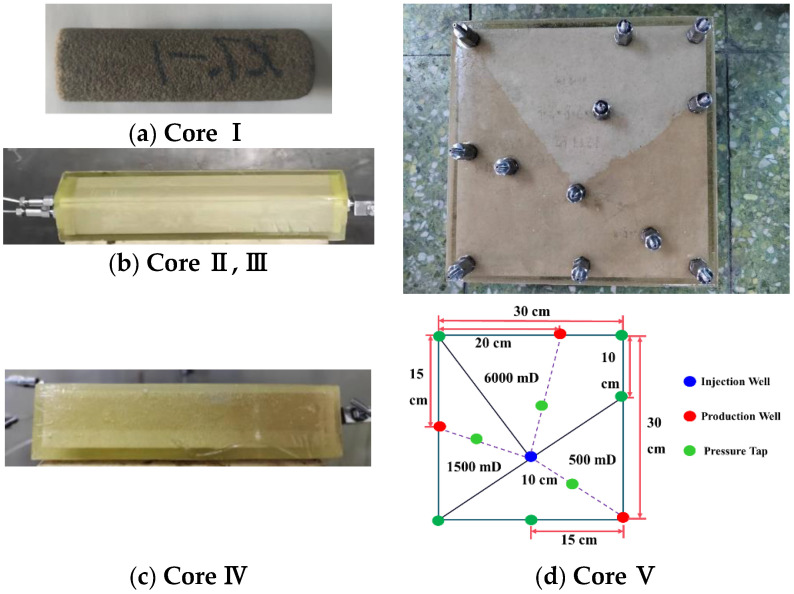
Physical and design drawings of cores.

The average permeability distribution range of the B oilfield block was 500–6000 mD, and the permeability ratio distribution was within the range of 2–10, mainly concentrated around 3–4. Based on the characteristics of the B oilfield block, artificial cores were designed, including four types, with parameters listed in Table 1. Among them, Core IV was a plane heterogeneous model scaled down from a field model according to similarity criteria for laboratory conditions. Physical and design drawings of the cores are shown in Figure 2.

#### 2.1.3. Experimental Fluid Physical Property Data

The oil used in the experiment was simulated oil with a viscosity of 20 mPa·s at 65 °C, and it belongs to light crude oil. The water used in the experiment was injection water from the block, with a mineralization degree of 7052.73 mg/L, and the ion composition is shown in Table 2.

The experimental equipment mainly included a viscometer, vacuum pump, core holder, displacement pump, pressure sensor, intermediate container, etc.

### 2.2. Experimental Methods

#### 2.2.1. Gel Formation Performance Test

A 10,000 mg/L mother liquor of the functional polymer was prepared, and a crosslinking agent was added in a ratio of functional polymer to crosslinking agent of 2:1, followed by dilution to different target concentrations. The viscosity changes over time were tested at different concentrations of the agents.

#### 2.2.2. Gel Injection Capacity and Plugging Rate Test

Core I was evacuated and saturated with water. Water flooding was conducted at 0.5 mL/min in a 65 °C incubator until the pressure stabilized, and the water flooding pressure was recorded. The designed functional polymer gel slug was injected, and the injection pressure was recorded to calculate the resistance coefficient. After aging for 7 days at reservoir temperature, subsequent water flooding was conducted until the pressure stabilized, and the injection pressure during the experiment was recorded to calculate the residual resistance coefficient and core plugging rate.

#### 2.2.3. Core Oil Displacement Experiment

The cores were evacuated, saturated with water, and their pore volumes were calculated. The cores were placed in a 65 °C incubator to simulate the formation environment and saturate them with oil, and the original oil saturation was calculated. Water flooding was conducted at 0.5 mL/min in the 65 °C incubator until the water content reached 90%, followed by chemical flooding to the designed slug. After aging for 7 days, water flooding was resumed until the water content reached 98%, and the experiment ended. Parameters such as pressure and oil–water production during the experiment were recorded, and the recovery degree was calculated. The experimental scheme design is shown in Table 3.

## 3. Results and Discussion

### 3.1. Gel Formation Performance Evaluation

**Figure 3:**
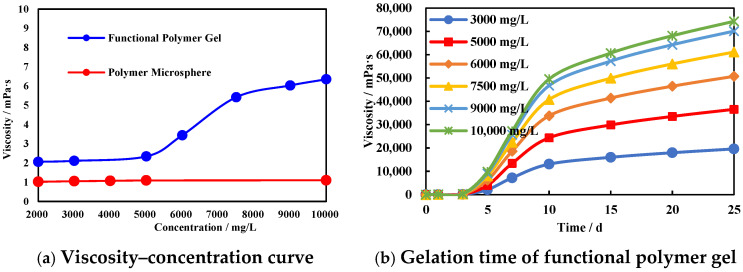
Viscosity curve of polymer agent.

The viscosity–concentration curves of the functional polymer gel and polymer microsphere are shown in the Figure 3. As shown in Figure 3a, the initial viscosities of both the functional polymer gel and the polymer microsphere were very low. For the functional polymer gel, within the concentration range of 2000–10,000 mg/L, the initial viscosities were always below 10 mPa·s. Within the concentration range of 2000–5000 mg/L, the initial viscosity fluctuated within 0.3 mPa·s and stabilized around 2 mPa·s. When the concentration exceeded 5000 mg/L, the initial viscosity began to increase and gradually stabilized after reaching a concentration of 7500 mg/L, ultimately stabilizing around 6 mPa·s. For the polymer microsphere, within the concentration range of 2000–10,000 mg/L, the initial viscosity fluctuated by no more than 0.08 mPa·s and remained stable at around 1 mPa·s.

The gel formation time of the functional polymer gel is shown in Figure 3b. The initial viscosities of the functional polymer gels were all below 10 mPa·s, and the initial gel formation times were all around 5 days. When the gel formation time was 7 days, the gel viscosity increased significantly. After the gel formation time exceeded 10 days, the gel viscosity increased slowly and gradually stabilized. When the functional polymer gel concentration was ≥7500 mg/L and the gel formation time was 7 days, the gel viscosity exceeded 20,000 mPa·s. After the gel formation time reached 20 days, the viscosity ranged from 56,000 to 74,500 mPa·s. The gel formation time and gel strength of the functional polymer gel are both approximately double those of the conventional gel. Based on this, the concentration of the functional polymer gel is determined to be 6000–9000 mg/L, with an aging time of over 7 days.

### 3.2. Testing of Gel Injection Capacity and Plugging Rate

The resistance factor (RF) and residual resistance factor (RRF) of the polymer agent at different concentrations and core permeabilities are shown in Table 4. Table 4 reveals that when the concentration of the polymer microsphere is 1000–5000 mg/L and the core permeability ranges from 500 to 2000 mD, the maximum RF is only 1.3, indicating low initial viscosity, good injectability in the core, and little difference from water injection flow. However, the maximum RRF can reach 5.6, and the maximum core plugging rate can reach 82.1%, suggesting that after aging, the polymer microsphere’s viscosity increases, exerting a certain plugging effect on the core pore throat and effectively improving water flooding channels. Under the same permeability conditions, as the agent concentration increases, so do the RF, RRF, and core plugging rate. Conversely, under the same agent concentration, as the core permeability increases, these factors decrease. Based on this, the concentration range for the polymer microsphere is determined to be 3000–5000 mg/L.

When the concentration of the functional polymer gel is 3000–9000 mg/L and the core permeability ranges from 2000 to 6000 mD, core permeability has little impact on the RF, which is mainly influenced by the agent concentration. At a concentration of 3000 mg/L, the RF is approximately 2; at 6000 mg/L, it is around 3.5; and at 9000 mg/L, it is nearly 6.5. Analysis suggests that the initial viscosity of the functional polymer gel is low, and the core permeability is high, leading to good agent injectability and thus a lower RF.

Under the same permeability conditions, as the agent concentration increases, the RF, RRF, and core plugging rate all increase. Conversely, under the same agent concentration, as the core permeability increases, these factors decrease. This analysis suggests that when the agent concentration increases, the gel strength increases, enhancing the plugging effect on the core. When the core permeability increases, the injectability and fluidity of the agent in the core improve, leading to earlier agent breakthrough, inefficient or ineffective injection, and no further increase in core plugging strength. Based on this, the concentration range for the functional polymer gel is determined to be 6000–9000 mg/L.

### 3.3. Agent Concentration Optimization

Experimental data on the impact of different agent concentrations on the oil recovery are shown in Table 5. Table 5 indicates that as the agent concentration increases, the growth rate of the oil recovery gradually slows down. Analysis suggests that when the agent concentration reaches a certain range, its performance also gradually plateaus, resulting in no significant changes during the oil displacement process. When the polymer microsphere concentration exceeds 4000 mg/L, it has little impact on the increase in the oil recovery. For the functional polymer gel, when the concentration exceeds 7500 mg/L, the gel strength meets the core plugging requirements, and the flow resistance generated by the functional polymer gel in high-permeability layers is sufficient to effectively develop low-permeability layers. Therefore, changes in gel strength have little impact on the oil recovery.

**Figure 4:**
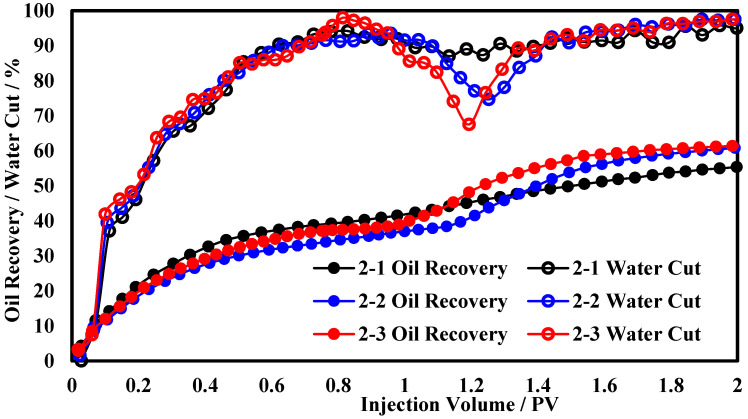
Production dynamic curves of polymer microsphere at different concentrations.

The production performance curves for different concentrations of the polymer microsphere are shown in Figure 4. As the concentration of the polymer microsphere increases, the oil recovery increases, but the rate of increase decreases. When the concentration exceeds 4000 mg/L, there is little difference in the oil recovery, which is approximately 6.4% higher than that at a concentration of 3000 mg/L. For the water cut curve, when the concentration exceeds 4000 mg/L, there is no significant change, with the lowest water cut being 67.5% and the duration below 90% water cut being 0.35 PV. For the polymer microsphere at a concentration of 3000 mg/L, the reduction in water cut is not significant, with the lowest water cut being only 87.3%. The analysis suggests that when the concentration of the polymer microsphere increases to 4000 mg/L, there is little variation in agent performance, leading to insignificant differences in oil displacement results. However, when the agent concentration is less than 4000 mg/L, there are substantial differences in agent performance, resulting in variations in production performance during the displacement process.

**Figure 5:**
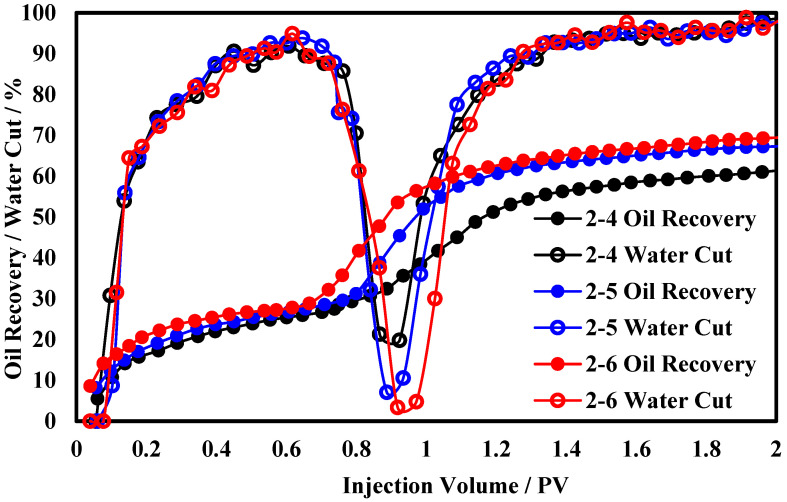
Production dynamic curves of functional polymer gel at different concentrations.

The production performance curves for functional polymer gels at different concentrations are shown in Figure 5. As the concentration of the functional polymer gel increases, the period of significant increase in the oil recovery advances, the oil production rate during the high-speed oil production phase gradually increases, and the rate of increase in the oil recovery diminishes. When the concentration exceeds 7500 mg/L, the oil recovery reaches above 67.3%, which is 6.2% higher than at a concentration of 6000 mg/L. As for the water cut curves, when the concentration of the functional polymer gel is greater than 7500 mg/L, the lowest water cut can be below 70%. At a concentration of 6000 mg/L, the lowest water cut is only 19.8%, with a difference of approximately 13% between the two. The analysis indicates that, when the concentration of the functional polymer gel reaches 7500 mg/L, its gel strength satisfies the plugging requirements for the high-permeability layer of the core, and the seepage resistance generated in the high-permeability layer is sufficient for the effective development of the low-permeability layer. Therefore, enhancing the gel strength by altering the gel concentration has a limited impact on the oil recovery.

### 3.4. Optimization of Chemical Slug Size

Table 6 presents experimental data on the impact of different chemical sizes on the oil recovery. Table 6 reveals that, as the chemical size increases, the rate of increase in the oil recovery gradually slows down. The analysis suggests that, due to the low initial viscosity and strong injectability of the two types of chemicals, they enter the core along the water-driving channels of the high-permeability layer during injection. When the injected size is too large, it can lead to direct production at the production end, resulting in the waste and ineffective injection of chemicals, which cannot further enhance the profile control effect and impact the oil recovery.

**Figure 6:**
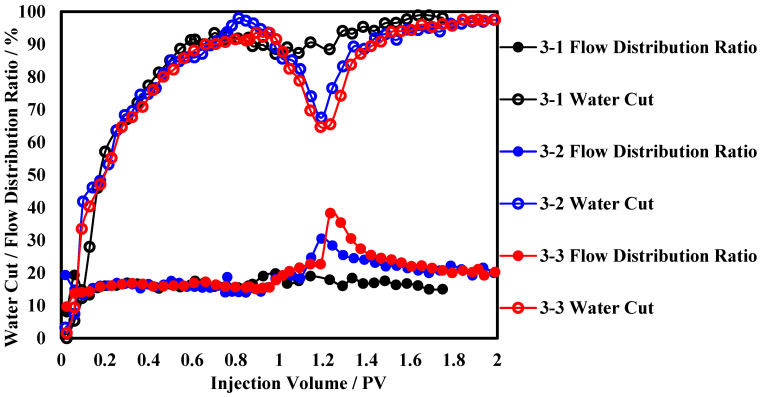
Production dynamic curves under different slug sizes of polymer microsphere.

The production performance curves of the polymer microsphere at different sizes are shown in Figure 6. The water cut curves indicate that there is little difference when the slug size is 0.3 PV and 0.5 PV, with the lowest water cut being approximately 67.5%. When the slug size is 0.1 PV, the lowest water cut is only 87.3%. Observing the flow distribution ratio curves, when the slug size is 0.1 PV, the polymer microsphere barely alters the flow distribution in the low-permeability layer. When the slug size is 0.3 PV, the flow distribution ratio in the low-permeability layer can rise to a maximum of 30.5% and then stabilize 22.2%. The analysis attributes this to the low initial viscosity of the agent. When the size is too large, the agent is produced along the high-permeability layer, resulting in low agent utilization efficiency. The continuous increase in slug size does not effectively increase the oil recovery again.

**Figure 7:**
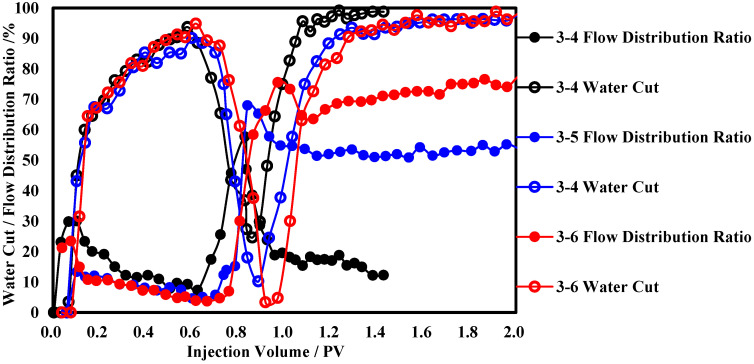
Production dynamic curves under different slug sizes of functional polymer gel.

The production performance curves during the displacement process of functional polymer gel at different sizes are shown in Figure 7. Observing the water cut curves, when the slug size is 0.05 PV, the plugging ability of the functional polymer gel for the high-permeability layer of the core is weak, with the smallest reduction in water cut compared to other slug sizes and the fastest recovery rate. When the slug size is greater than 0.1 PV, the water cut can drop below 10%, with similar degrees of reduction and a stable time below 90% water cut of approximately 0.6 PV. Observing the flow distribution ratio curves of the low-permeability layer, when the slug size is 0.05 PV, the flow distribution ratio in the low-permeability layer rises rapidly and then falls rapidly, reaching a maximum of only 57.7% and eventually stabilizing around 17%. When the slug size is greater than 0.1 PV, the flow distribution ratio in the low-permeability layer rises rapidly and stabilizes above 50%. The analysis suggests that there is a correlation between the agent size and the core plugging rate. When the agent size increases to 0.1 PV, the gel plugging strength is sufficient for the development of the low-permeability layer, so further increasing the agent size has little impact on the oil recovery.

### 3.5. Study on Oil Displacement Effect in Different Heterogeneous Models

#### 3.5.1. Study on Oil Displacement Effect in Vertically Heterogeneous Models

**Figure 8:**
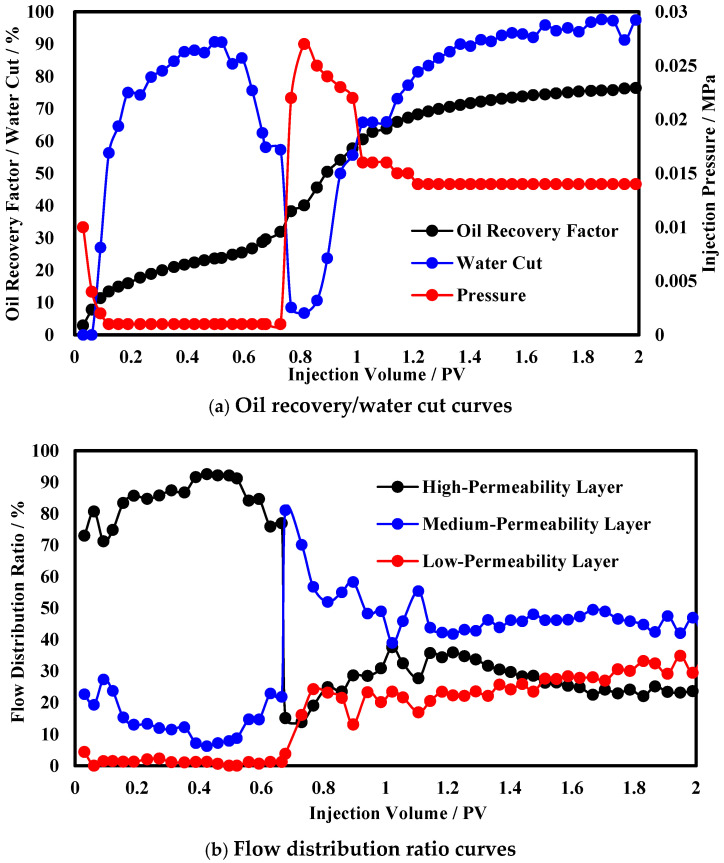
Production dynamic curves in heterogeneous reservoirs.

To simulate the vertical development effect of the reservoir, a three-layer heterogeneous core IV was designed. Figure 8a shows the production performance curves during the three-layer heterogeneous oil displacement process. The oil recovery by water flooding is 23.8%, and the final oil recovery can reach 76.8%, which is a 53% increase compared to the oil recovery by water flooding. The water cut reaches 90% at 0.5 PV of water flooding and drops to a minimum of 6.7% after chemical profile control, with a displacement time below 70% water cut of 0.55 PV. The pressure does not differ significantly between the functional polymer gel injection stage and the water flooding stage. After gel formation, the injection pressure of the polymer microsphere suddenly rises to 0.027 MPa and then gradually decreases, stabilizing at 0.014 MPa. The analysis suggests that the initial viscosity of the functional polymer gel is low, resulting in low injection pressure. After gel formation, the high-permeability layer is plugged, causing an increase in displacement pressure, a decrease in water cut, and a significant increase in the oil recovery.

The flow distribution ratio curves during oil displacement in heterogeneous models are shown in Figure 8b. During the water flooding process, the flow distribution ratio of the high-permeability layer can reach 90%, the middle-permeability layer decreases from 30% to 7%, and the utilization rate of the low-permeability layer is low, with the flow distribution ratio of the low-permeability layer remaining below 3% throughout the water flooding process. After chemical profile control, the flow distribution ratio of the high-permeability layer fluctuates around 30%; the middle-permeability layer’s flow distribution dominates, subsequently stabilizing around 45%; and the low-permeability layer is also effectively developed, with the flow distribution ratio stabilizing around 25%. The analysis attributes this to the plugging of the high-permeability layer by the functional polymer gel, which increases the seepage resistance of the high-permeability layer. During the subsequent injection of the polymer microsphere, the seepage resistance of the middle-permeability layer gradually increases, leading to a gradual increase in the flow distribution ratio of the low-permeability layer.

#### 3.5.2. Study on Oil Displacement Effect in Planar Heterogeneous Models

**Figure 9:**
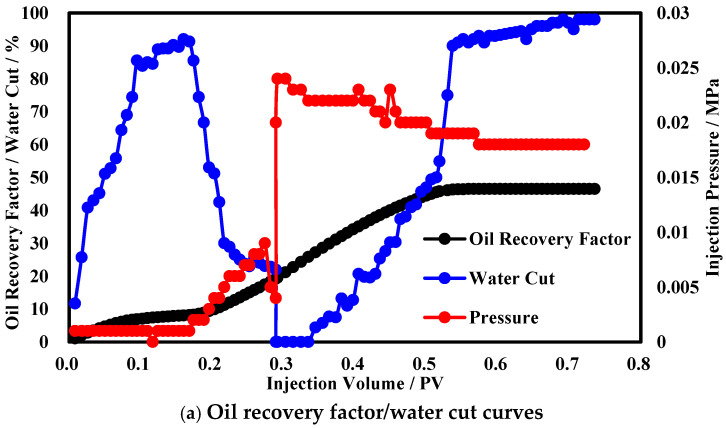

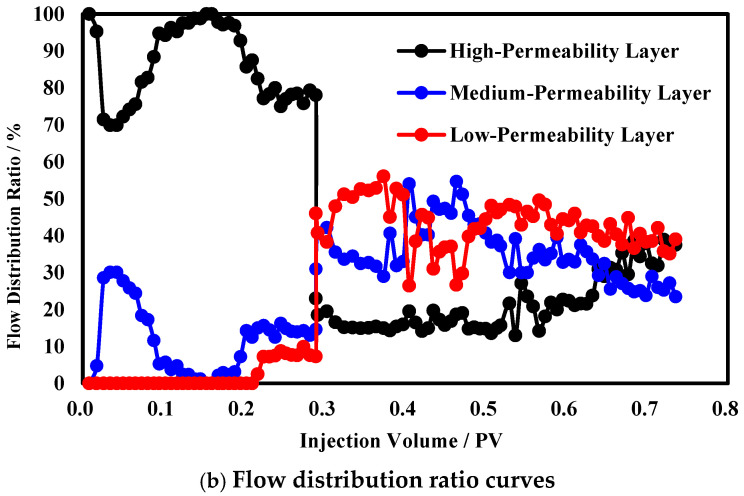
Production dynamic curves in planar heterogeneous reservoirs.

To simulate reservoir development performance, a planar heterogeneous core V was designed, and the production dynamic curves are shown in Figure 9a. During the water flooding stage, the water cut rose rapidly, reaching 90% around 0.17 PV, with a water flooding recovery factor of only 7.8%. During the functional polymer gel injection stage, due to the long injection time, the functional polymer gel properties gradually changed during injection, exerting a certain profile control function. The injection pressure rose, and the water cut rapidly dropped to 27%, before its profile control capacity reached its limit and the water cut stabilized. In the subsequent stage, pressure surged, and the water cut plummeted, with a 0.03 PV period of oil production without water. Afterward, pressure slowly declined, the water cut steadily rose, and the recovery factor continued to increase to 46%. After a cumulative injection of 0.5 PV, the water cut surged again, pressure flattened out, and the recovery factor stabilized at 46.5%.

The diversion rate curve of agents during the planar heterogeneous core displacement process is shown in Figure 9b. After profile control, the diversion rate of the high-permeability layer could stabilize at around 15% for about 0.2 PV, before the diversion rate of the high-permeability layer began to slowly rise, reaching 37.5% at the end of displacement. After profile control, the diversion rates of the medium- and low-permeability layers fluctuated more significantly, with the overall diversion rates of the medium- and low-permeability layers fluctuating around 45%. Analysis suggests that when the functional polymer gel was injected, it blocked along the high-permeability water flooding channels, while a small amount of low initial viscosity agent entered the medium- and low-permeability layers, causing slight blockage of the reservoir. Subsequently, the polymer microsphere was injected, and the diversion rate of the low-permeability layer dominated. After the blockage of the medium-permeability layer was broken through, the diversion rates of the medium- and low-permeability layers began to fluctuate because of the plugging and migration of the polymer agent in complex reservoirs.

**Figure 10:**
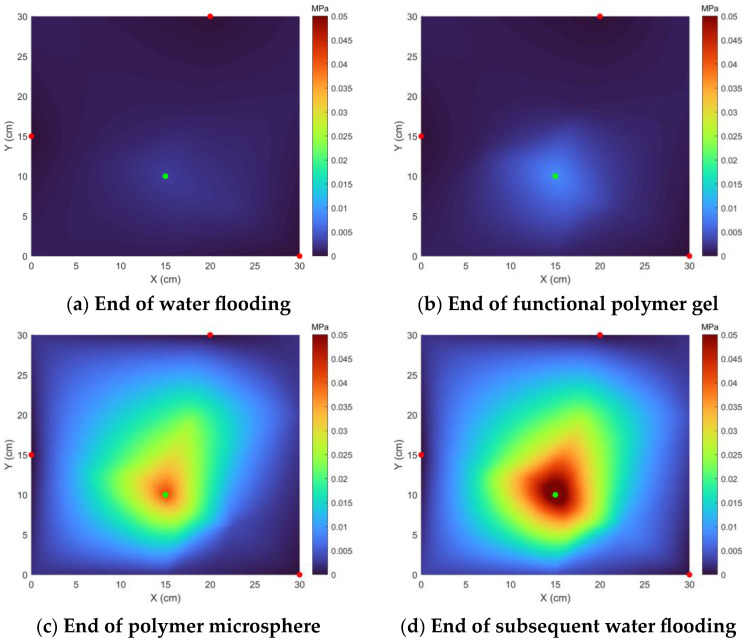
Planar distribution map of pressure. (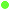 Injection Well; 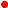 Production Well).

Pressure field diagrams at different stages are shown in Figure 10. Analysis reveals that in Figure 10a, after the water flooding ended, pressure distribution was relatively uniform throughout the core, but careful study found that the pressure gradient was smallest along the mainstream direction in the high-permeability area. Analysis suggests that sandstone has high permeability and low crude oil viscosity, resulting in low water flooding pressure, and water flows away along high-permeability channels, with small pressure fluctuations throughout the core. In Figure 10b, after functional polymer gel ended, pressure around the injection well rose, and pressure in the high-permeability channels rose significantly. Analysis suggests that the functional polymer gel had low initial viscosity and was injected along high-permeability channels, with a viscosity increase phenomenon during injection that exerted a certain profile control function. At this time, high blockage had not yet formed, so pressure changes in the entire area were small. In Figure 10c, after polymer microsphere ended, the pressure gradients of the mainstream directions in the three areas were high-permeability layer > medium-permeability layer > low-permeability layer. Analysis suggests that the profile control agent had blocked the high-permeability layer, and the polymer microsphere was gradually accumulating in the medium-permeability layer, while effectively exploiting the medium-permeability layer, resulting in a higher pressure gradient in the medium-permeability area. In Figure 10d, after the subsequent water stage ended, it was clear that the pressure gradient in the mainstream direction of the medium- and high-permeability areas was higher than that in the low-permeability area. Analysis suggests that the functional polymer gel and polymer microsphere were functioning, blocking the medium- and high-permeability layers, while effectively exploiting the low-permeability layer at this time. Analysis of the entire process suggests that this scheme achieved gradual exploitation of high-, medium-, and low-permeability layers, with effective exploitation.

## 4. Conclusions

This study, based on offshore sandstone reservoirs, conducted performance evaluations of functional polymer gels and polymer microspheres. Through a series of heterogeneous core flooding experiments, the chemical flooding scheme was optimized and designed, yielding the following conclusions:

(1) The initial viscosities of the functional polymer gel and polymer microsphere are both below 10 mPa·s. When the concentration of the functional polymer gel reaches 6000 mg/L, the plugging rate for a core with a permeability of 6000 mD can exceed 90%. Similarly, when the concentration of the novel polymer microsphere reaches 3000 mg/L, the plugging rate for a core with a permeability of 2000 mD can reach over 80%.
(2) As the agent concentration increases, the increase in oil recovery gradually flattens out. Due to the influence of the static properties of the agents and the nature of the cores, it is recommended to use the functional polymer gel concentration of 7500 mg/L and the polymer microsphere concentration of 4000 mg/L. Increasing the agent concentration further does not effectively enhance the profile control.
(3) With the increase in slug size, the increase in oil recovery also gradually flattens out. Considering the initial viscosity of the agents and the nature of the cores, it is recommended to use the functional polymer gel slug size of 0.1 PV and the polymer microsphere slug size of 0.3 PV. Increasing the slug size further may lead to breakthroughs of the agents from the high-permeability layers, resulting in inefficient utilization.
(4) During the development of vertically heterogeneous reservoirs, the combined approach of functional polymer gel and polymer microsphere is adopted. The functional polymer gel plugs the high-permeability layers, while the polymer microsphere retains in the medium-permeability layers, increasing the diversion rate of the low-permeability layers. This achieves the goal of gradual utilization of multiple layers and improves oil recovery.
(5) In the development of planar heterogeneous reservoirs, the combined approach of functional polymer gel and polymer microsphere alters the order of pressure gradients in different regions at different stages, effectively exploiting areas with different permeabilities and enhancing oil recovery.

The current study has not yet investigated the mechanical properties of functional polymeric gels and polymeric microspheres, nor their microscopic oil displacement mechanisms. Subsequent research will focus on these two aspects to deepen the understanding of how polymer agents enhance oil recovery in complex reservoirs.

## Figures and Tables

**Table 1:** Core design parameters.

No.	Size	Gas Permeability	Remarks
Core I	Φ2.5 × 10	500, 1000, 2000, 4000, 6000 mD	One-dimensional cylindrical core for testing core plugging rate
Core II	4.5 × 4.5 × 30 cm	1500/500 mD	Two-layer heterogeneous, one injection and two productions
Core III	4.5 × 4.5 × 30 cm	6000/2000 mD	Two-layer heterogeneous, one injection and two productions
Core IV	4.5 × 6.75 × 30 cm	6000/1500/500 mD	Three-layer heterogeneous, one injection and three productions
Core V	4.5 × 30 × 30 cm	6000/1500/500 mD	Plane heterogeneous, one injection and three productions

**Table 2:** Mineral composition of simulated water.

Ionic Composition	K^+^, Na^+^	Ca^2+^	Mg^2+^	CO_3_^2−^	HCO_3_^−^	SO_3_^2−^	Cl^−^	Total
Content/mg/L	2323.97	171.54	21.82	56.12	825.39	12.6	3641.29	7052.73

**Table 3:** Experimental scheme design.

No.	Core Type	Agent Type	Agent Concentration (mg/L)	Slug Size (PV)
2-1~3	Core II	Polymer microsphere	3000, 4000, 5000	0.3
2-4~6	Core III	Functional polymer gel	6000, 7500, 9000	0.15
3-1~3	Core II	Polymer microsphere	4000	0.1, 0.3, 0.5
3-4~6	Core III	Functional polymer gel	9000	0.05, 0.1, 0.15
4-1	Core IV	Agent combination	7500 mg/L Functional polymer gel 0.1 PV + 4000 mg/L Polymer microsphere 0.3 PV	
4-2	Core V			

**Table 4:** Resistance factor and residual resistance factor of polymer agent at different concentrations and core permeabilities.

No.	Agent Type	Concentration mg/L	Permeability mD	RF	RRF	Core Plugging Rate
I	Polymer microsphere	1000	500	1	3.2	68.5
			1000	1	2.8	64.7
			2000	1	2.6	61.3
		3000	500	1.2	4.2	76.4
			1000	1	3.7	73.2
			2000	1	3.4	70.4
		5000	500	1.3	5.6	82.1
			1000	1.3	5.3	81.3
			2000	1.2	5	80.1
II	Functional polymer gel	3000	2000	2.3	11.5	91.3
			4000	2	8.8	88.6
			6000	2	6	83.4
		6000	2000	3.7	23.3	95.7
			4000	3.5	16.9	94.1
			6000	3.4	11.5	91.3
		9000	2000	6.7	41.7	97.6
			4000	6.5	31.3	96.8
			6000	6	20.8	95.2

**Table 5:** Oil recovery factor at different concentrations of agent.

No.	Core Type	Agent Type	Concentration mg/L	So %	Oil Recovery/%		EOR %
					Water Flooding	Final	
2-1	Core II	Polymer microsphere	3000	68.7	39.3	56.7	17.4
2-2	Core II		4000	70.1	37.5	61.3	23.8
2-3	Core II		5000	68.2	38.5	62.6	24.1
2-4	Core III	Functional polymer gel	6000	71.4	27.5	61.5	34
2-5	Core III		7500	73.0	28.8	67.3	38.5
2-6	Core III		9000	73.1	29.5	69.5	40

**Table 6:** Oil recovery factor at different slug sizes of chemicals.

No.	Core Type	Agent Type	Agent Size PV	So %	Oil Recovery/%		EOR %
					Water Flooding	Final	
3-1	Core II	Polymer microsphere (4000 mg/L)	0.1	68.7	36.8	48.5	11.7
3-2	Core II		0.3	70.1	37.5	61.3	23.8
3-3	Core II		0.5	69.2	37.4	62.7	25.3
3-4	Core III	Functional polymer gel (9000 mg/L)	0.05	71.3	28.7	56.3	27.6
3-5	Core III		0.1	73.6	29.3	67.8	38.5
3-6	Core III		0.15	73.1	29.5	69.5	40

## Data Availability

The original contributions presented in this study are included in the article. Further inquiries can be directed to the corresponding author.
